# Interventions supporting people from Black, Asian and Minority Ethnic groups in the United Kingdom with musculoskeletal disorders: a scoping review

**DOI:** 10.3389/fpubh.2025.1675860

**Published:** 2025-10-02

**Authors:** Nasreen Ali, Zahra Maryam, Srinivasan Thanigachalam, Pei-Ching Tseng, Fani Liapi, Nishani Jeyapalan, Yannis Pappas, Gurch Randhawa, Britzer Paul Vincent

**Affiliations:** ^1^Institute for Health Research, University of Bedfordshire, Luton, United Kingdom; ^2^AIIMS, Madurai, India

**Keywords:** scoping review, musculoskeletal disorders, ethnic minority, “Black, Asian, and Minority Ethnic”, United Kingdom

## Abstract

**Background:**

Musculoskeletal disorders (MSDs) significantly impact quality of life, particularly among Black, Asian, and Minority Ethnic communities in the UK, who face disproportionate burdens and barriers to care. Despite growing recognition of ethnic health disparities, there is limited understanding of interventions tailored to these populations. This scoping review aimed to map the existing literature on interventions for MSDs among Black, Asian, and Minority Ethnic groups in the UK, identifying key approaches, gaps, and opportunities for culturally appropriate healthcare delivery.

**Method:**

A scoping review was conducted following the Population–Concept–Context (PCC) framework. Seven databases (PubMed Central, CINAHL, Scopus, Medline Full-text, Web of Science, PsycInfo, and Cochrane) were systematically searched up to April 2025. Studies were included if they assessed MSD interventions among Black, Asian and Minority Ethnic individuals in the UK. Both qualitative and quantitative studies were considered. Data were synthesised narratively using thematic analysis supported by NVivo v.11 software.

**Findings:**

Nine studies met the inclusion criteria. Three primary themes emerged: (1) language and communication initiatives, such as multilingual resources and telephone helplines; (2) pharmacological and dietary interventions, particularly addressing vitamin D deficiency and rheumatoid arthritis management; and (3) peer-support and educational initiatives, including community-based and culturally tailored programmes. A significant underrepresentation of Black African and Black Caribbean populations was identified alongside a noticeable lack of participatory or qualitative research approaches.

**Conclusion:**

There is a critical need for ethnically inclusive, culturally tailored MSD interventions in the UK. Future research should prioritise holistic, community-based approaches and actively address structural inequalities to improve health outcomes and ensure equitable care across all ethnic groups.

## Introduction

Musculoskeletal Disorders (MSDs) are conditions that impact the body’s movement or musculoskeletal system, including bones, joints, muscles, tendons, ligaments, nerves, and other supporting tissues (1). Musculoskeletal (MSK) conditions encompass a wide range of diagnoses, including arthritis, bone and joint conditions, congenital conditions, maxillofacial conditions, soft tissue conditions, and spinal and back conditions (1). In developed countries, the prevalence of MSDs varies across different ethnic groups (2, 3) and is particularly common among older adults; thus, life expectancy increases the overall burden of MSDs and is expected to rise correspondingly (4).

UK-focused studies of MSDs have reported a higher prevalence among the Black, Asian, and Minority Ethnic groups compared to their White British counterparts (5). For example, research has shown a greater incidence of vitamin D deficiency-related bone disorders such as rickets and osteomalacia within South Asian communities. These disparities are largely attributed to cultural, dietary, and environmental factors (6, 7). Evidence also highlights that MSK pain is more widespread among Black, Asian, and Minority Ethnic groups, which may reflect social, cultural, and psychological differences (5).

Black, Asian, and Minority Ethnic groups in the UK also face distinct challenges in the management of MSDs. Compared to the White British population, they experience greater levels of disability, reduced access to specialist care, diagnostic delays, barriers to effective treatment, and underutilisation of services (8–11). These disparities are influenced by a range of intersecting factors, including language and communication barriers, differing cultural health beliefs, socioeconomic disadvantage, and systemic bias within healthcare systems (9, 12).

Despite well-documented disparities, there remains a significant gap in research focused on effective interventions tailored to the needs of Black, Asian, and Minority Ethnic groups with MSDs in the UK. While various interventions have been evaluated for both White British and Black, Asian, and Minority Ethnic populations, no comprehensive review has systematically mapped existing data specific to interventions designed for minoritised groups using an evidence synthesis method (13).

Recent evidence highlights that a key gap in evidence is the lack of culturally appropriate interventions tested specifically within ethnic minority populations, as most chronic illness self-management strategies are based on research involving white British groups (14). A scoping review is therefore needed to identify studies that have assessed interventions for MSDs among Black, Asian, and Minority Ethnic groups in the UK. Such a review can be instrumental in informing the development of targeted, culturally appropriate strategies to address health inequalities. By mapping existing research, this scoping review provides valuable insights into what works, highlighting critical gaps in the evidence base, and supports the design of future initiatives aimed at improving the health and well-being of people from Black, Asian, and Minority Ethnic backgrounds living with MSK conditions (13). This scoping review aimed to map the existing literature on MSD interventions for Black, Asian, and Minority Ethnic groups in the UK, identifying key approaches, gaps, and opportunities to strengthen culturally appropriate healthcare delivery. We undertook a scoping review to synthesise the literature on different interventions among Black, Asian, and Minority Ethnic groups in the UK who have MSDs (13).

## Methods

### Study design

Scoping reviews are particularly beneficial for mapping the breadth and nature of evidence on a broad topic, identifying key concepts, gaps, and types of available research without assessing study quality (13, 15). In contrast to systematic reviews, which focus on answering specific, narrowly defined questions with strict methodological criteria, scoping reviews provide a more flexible approach suited for emerging or complex areas of study (16). This strong and comprehensive evidence base further informs a wide range of primary research that in turn could influence the practice (15, 16).

### Scoping review framework

The framework proposed by Arksey and O’Malley has been highly influential in the development of scoping review methodology (13). Following this guideline, Levac et al. later refined this framework, providing more detailed guidance for each stage of the review process, thereby enhancing its clarity and methodological rigour (17). However, these two frameworks were studied in detail to form the foundation of the Joanna Briggs Institute (JBI) approach to scoping reviews (15). Given this robust and well-established methodological base, the JBI methodology was adopted for the conduct of the present scoping review (15).

We followed the ‘Population-Concept-Context’ (PCC) framework for this scoping review (15), and this runs through the scoping review question, search strategy, and inclusion and exclusion criteria. The PCC framework is preferred in scoping reviews because it supports broad, exploratory questions rather than narrowly focused ones. It accommodates diverse evidence types, including qualitative studies, without requiring a specific intervention or comparison (15). It also emphasises ‘*context’*, which is essential for understanding complex issues beyond simple cause–and–effect relationships, particularly in this review.

### Search strategy

Seven databases, PubMed Central, CINAHL, Scopus, Medline Full-text, Web of Science, Psyc Info, and Cochrane, were searched using the search strategy until the end of April 2025. The choice of databases and search terms was made in consultation with an experienced subject librarian (DA). The databases were searched with a combination of search terms (MESH terms, Subject Headings, Major Headings, Title-Abstract-Keywords) using the Population, Concept, and Context (PCC) framework (15). For instance, Population: Black, Asian, and Minority Ethnic groups, Concept: Interventions, Context: United Kingdom. Details on the search strategy are available in Supplementary data S1.

### Inclusion and exclusion criteria

To be considered for this review, studies were required to meet the following criteria:

1. *Population*: Studies were eligible if they focused on individuals from Black, Asian, and Minority Ethnic groups in the UK. Eligible participants included individuals who were diagnosed with MSDs and their family members. Each study had to assess an intervention and include participants from Black, Asian, and Minority Ethnic groups living in the UK. Where other major ethnic groups in the UK were also involved, only studies that presented separate findings specific to Black, Asian, and Minority Ethnic populations were considered.
2. *Concept*: Studies were included if they assessed interventions related to MSDs.
3. *Context*: Only studies conducted in the UK, peer-reviewed, available in English and with access to full-text were included.

Studies that combined the findings of Black, Asian, and Minority Ethnic groups with other major ethnic groups in the UK were excluded from the review. Though this is a scoping review, we did not explore the views/perspectives from healthcare professionals. Additionally, conference posters, conference presentations, books and book chapters, unpublished literature, grey literature, editorials, and commentaries were excluded from the review.

### Screening

**Figure 1:**
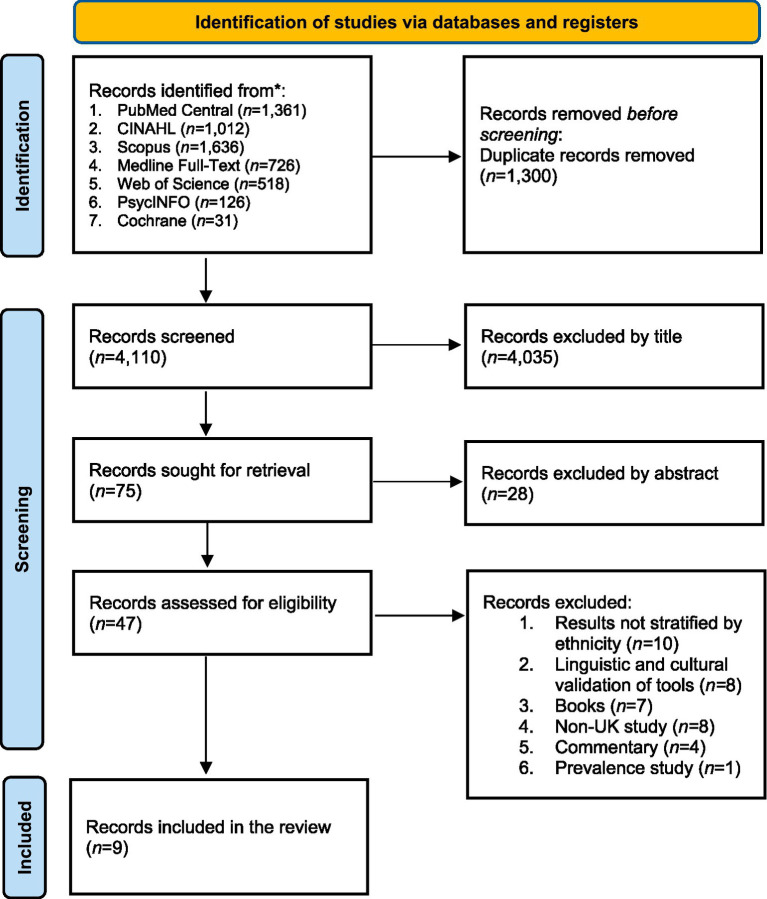
PRISMA flowchart.

A total of 5,419 were retrieved from seven databases (see Figure 1). These were exported to Rayyan software,^1^ where 1,300 duplicate records were removed. The remaining 4,110 studies underwent double-blinded screening. Discrepancies arose between the two primary reviewers (ZM and ST) regarding 20 studies, which were resolved through discussion with a third independent reviewer (BPV). Following title and abstract screening, 47 studies were selected for full-text review by two primary reviewers (ZM and ST). Of these, results were not stratified by ethnicity for 10 studies, 8 of the studies were linguistic and cultural validation of scales, 7 were books, 7 were non-UK studies, 4 were commentaries, and 1 was a prevalence study (see Figure 1). Finally, 9 studies met the eligibility criteria and were included in the final review (see Figure 1), with data extraction carried out by three reviewers (BPV, NA, and ZM).

### Data synthesis

The narrative synthesis approach outlined by Popay et al. was employed to synthesise data extracted from both quantitative and qualitative studies (18). This method allowed the integration of findings across different study designs within a review. The process followed four key stages: (1) conducting a systematic search, (2) organising and clustering the included studies, (3) providing a textual description of each study, and (4) assessing and interpreting the findings. Three reviewers were involved in data synthesis (BPV, ZM, ST). Initially, we extracted information from the 9 studies to draft the evidence table (Table 1). Following this, we drafted a narrative summary for each included study, incorporating both quantitative and qualitative results. These summaries were then imported into NVivo V.11 software (Lumivero, 2015)^2^ for thematic coding. During interpretation, we examined where studies showed similar or differing findings. For any differing findings, we looked more closely at study details such as participant demographics and the type of interventions to identify possible reasons for the differences. Finally, we grouped the studies by type of intervention to produce a structured narrative summary of the findings.

**Table 1:** Evidence table.

Author et al (YYYY)	Location in the UK	Intervention type - themes	Aim	Research method	Sample size	Data collection method
Kumar et al., 2020 (19)	West Midlands	Theme 1	To explore how patients of South Asian origin make sense of their disease after receiving written leaflets compared with online information or visualising real-time Doppler US. images of their inflamed joints.	Qualitative research.	20	Face-to-face semi-structured interviews.
Kumar et al., 2010 (20)	Birmingham	Theme 1 & 3	To test the utility of educational resources provided by Birmingham Arthritis Resource Centre, including both the educational support session with a trained patient volunteer and the bilingual educational audio CD for patients of South Asian origin with rheumatoid arthritis (RA) who find it difficult to communicate in English.	Qualitative research.	15	Focus group discussion.
Kumar et al., 2009 (21)	Birmingham	Theme 1	To investigate what proportion of patients attending a rheumatology unit in Birmingham, UK, require interpretation services and to assess the use of an Asian language telephone helpline for those who find it easier to communicate in Punjabi, Urdu or Hindi than in English.	Quantitative research.	512	Survey.
Dunningan et al., 1981 (22)	Glasgow	Themes 2 & 3	To evaluate the effectiveness of a multidisciplinary, community-based vitamin D supplementation campaign in reducing the prevalence and severity of rickets and osteomalacia among the Asian population in Glasgow.	Quantitative research.	165	Survey.
Ford et al., 1972 (23)	NA	Theme 2	To evaluate the rachitogenic properties of unleavened bread in the Pakistani and Indian diet by substituting leavened bread with lower extraction factors.	Quantitative research.	10	Survey.
Pietrek et al., 1976 (24)	Glasgow	Theme 2	To assess and compare the effectiveness of weekly vitamin D₂ supplementation versus vitamin D-fortified chapatti flour in improving serum 25-hydroxy-vitamin D levels among Asian families living in Glasgow.	Quantitative research.	64	Survey.
Zahir Hussain et al., 2021 (25)	Leicester	Theme 2	To see whether there is any difference in baricitinib Disease Activity Score 28 (DAS28) response between the Asian and White cohorts	Quantitative research.	120	Survey.
Russell et al., 2024 (26)	United Kingdom (National cohort)	Theme 2	To investigate factors associated with biological and targeted synthetic DMARD initiation in a national cohort of patients with early rheumatoid arthritis within a universal health-care system.	Quantitative research.	6,098	Survey.
Wilson et al., 2024 (27)	London	Theme 3	To determine the feasibility of a community-based aquatic exercise and peer support intervention for patients with musculoskeletal disorders delivered via a multisector social enterprise that creates personalised exercise programmes delivered through an application on a tablet computer.	Mixed method.	4	Survey and interviews.

Theme 1, language and communication initiatives; Theme 2, pharmacological and dietary interventions; Theme 3, peer-support and educational initiatives.

## Findings

This scoping review included nine studies examining interventions aimed at improving MSDs, which included participants from Black, Asian and Minority Ethnic groups living in the UK. The studies were categorised into three primary themes based on the type of interventions tested: (1) language and communication interventions, (2) pharmacological and dietary interventions, and (3) peer-support and educational interventions.

### Language and communication interventions

Three studies (19–21) investigated interventions designed to overcome language and communication barriers faced by South Asian patients with rheumatoid arthritis (RA). These included printed leaflets, online resources, bilingual educational audio CDs, and a multilingual telephone helpline. One study (19) assessed the change in the knowledge among patients towards MSDs through leaflets and online material, which was moderately helpful when combined with face-to-face consultations. However, the non-availability of translated materials significantly limited their utility for non-English speakers. In contrast, Doppler ultrasound imaging emerged as a highly engaging tool. It enabled real-time visualisation of inflammation for those who have inflammatory MSD and engaged discussion with the healthcare team. This not only improved patients’ understanding of the disease but also motivated behavioural change and improved adherence to treatment, which could be used as an effective aid for Black, Asian, and Minority Ethnic individuals with MSD for improving communication (19).

To address literacy and language challenges, another study introduced a bilingual educational compact disc (CD) (20) recorded in Punjabi, Urdu, and Hindi. Patients found the audio format empowering, as it allowed repeated listening and sharing with family members, thereby promoting collective learning. While culturally and linguistically accessible, the CD lacked interactive capacity, which limited its ability to address individual concerns in real-time (20).

To overcome this gap, another study implemented a South Asian language telephone helpline (21). This service offered real-time communication in native languages and was especially beneficial for older adults and women with limited English proficiency. It provided emotional reassurance, improved understanding of RA and medication use, and fostered confidence in navigating health services. Taken together, these studies (19–21) show that while static resources such as leaflets and audio CDs offer foundational knowledge, interactive and culturally sensitive approaches like Doppler Ultra Sound (US), alongside discussion with healthcare teams and helplines, respectively, are more effective in empowering linguistically diverse populations.

### Pharmacological and dietary interventions

Five studies (22–26) evaluated the impact of pharmacological and dietary interventions on vitamin D deficiency, rickets, and rheumatoid arthritis, particularly in South Asian communities. Two studies explored the role of diet in vitamin D deficiency and bone disease. One study (23) found that South Asian patients who continued consuming chapattis, a staple rich in phytates, showed little biochemical improvement despite receiving vitamin D supplementation. Phytates interfered with calcium absorption, effectively neutralising the impact of supplementation. Conversely, patients who adopted a chapatti-free diet while receiving vitamin D and calcium therapy exhibited significant improvement in serum calcium and phosphate levels (23).

Complementing these findings, a second study (24) highlighted the multifactorial causes of vitamin D deficiency, including low sun exposure, poor dietary intake, and reliance on phytate-rich foods. It recommended dietary diversification and targeted supplementation. These findings confirm that pharmaceutical treatment must be paired with dietary modifications to be effective in this population (24). The Glasgow Rickets Campaign (22) supported that low-dose vitamin D supplementation via food fortification was inadequate for preventing rickets in South Asian children. In contrast, targeted supplementation with higher doses (≥400 IU/day) led to significant reductions in disease incidence, establishing a clear dose–response effect (22).

In terms of drug-based interventions for RA, one study evaluated the safety and efficacy of baricitinib across ethnic groups (25). It reported no significant differences in clinical response or adverse events between minority ethnic groups (the term used within the study) and White patients. However, small sample sizes of ethnic minorities limited broader generalisations, and further research was recommended (25). A national cohort study (26) revealed disparities in the initiation of advanced RA therapies. South Asian and Black British patients were less likely to receive biologic or targeted synthetic disease-modifying antirheumatic drugs compared to White patients. These disparities persisted even after adjusting for disease severity, indicating underlying structural barriers such as delayed referrals, language obstacles, and socioeconomic deprivation.

Together, these findings (22–26) emphasise the need for culturally tailored, multi-modal interventions that combine dietary education with equitable access to drug therapies, ensuring that Black, Asian and Minority Ethnic patients receive effective and timely care.

### Peer-support and educational interventions

Three studies (20, 22, 27) emphasised the benefits of peer support and educational strategies in managing MSDs among Black, Asian and Minority Ethnic groups living in the UK. A community-based service evaluation (27) examined an aquatic exercise and peer-support programme designed for individuals with musculoskeletal disorders. The intervention was both feasible and well-received. Participants reported increased confidence, better symptom management, and enhanced social inclusion (27).

Similarly, another intervention (20) used bilingual peer educators alongside an educational CD for South Asian RA patients. This culturally aligned, peer-delivered support improved disease understanding and medication adherence, while also facilitating discussions with family members, enhancing collective awareness (20).

The Glasgow Rickets Campaign (22) also employed peer educators as part of a culturally sensitive community outreach strategy alongside their drug and diet interventions. This approach contributed to improved awareness and uptake of vitamin D supplementation, particularly in South Asian communities. Peer-led initiatives helped convey complex health messages in familiar terms, thereby increasing trust and engagement (22).

Collectively, these studies (20, 22, 27) highlight the value of peer support embedded in culturally tailored educational programmes. Such interventions are especially effective when delivered in community settings, reinforcing the importance of trust, language familiarity, and cultural sensitivity in improving health outcomes for Black, Asian and Minority Ethnic populations living in the UK.

## Discussion

This scoping review highlighted a notable underrepresentation of Black, Asian and Minority Ethnic communities, along with a lack of clearly differentiated findings by ethnic background in MSD intervention research, despite the disproportionate burden of these conditions among such populations in the UK. While the reviewed literature offers valuable insights into communication, pharmacological, dietary, and peer-support strategies, the volume and scope of ethnically tailored research remain limited (14). This imbalance has implications for clinical effectiveness, health equity, and long-term policy planning (14).

A recurring theme throughout the studies is the underrepresentation of Black African and Black Caribbean populations. While South Asian communities were relatively well-represented (19–23), particularly concerning vitamin D deficiency and rheumatoid arthritis (22–24), there was minimal evidence from individuals of Black Caribbean or Black African origin (26). This is concerning given that research indicates Black Caribbean and Black African populations also face a disproportionately high burden of MSDs, such as osteoarthritis and back pain, and often report worse outcomes, including greater pain severity and disability (28, 29). Yet, targeted interventions and culturally sensitive healthcare responses remain largely underdeveloped for these communities.

Furthermore, while clinical studies are important, studies on communication strategies or community-based education are highly important among Black, Asian, and Minority Ethnic groups in the UK to improve access to care. However, in this review, we found that five of the nine studies addressed medical management, such as vitamin D supplementation, dietary modification, or RA drug therapy (22–26). While these studies provide valuable evidence, they reflect a clinical bias in intervention research, often overlooking psychosocial and behavioural determinants of health, which are very important for Black, Asian, and Minority Ethnic groups in the UK. For instance, only five studies addressed communication strategies or community-based education (19–22, 27), which are particularly relevant for Black, Asian and Minority Ethnic groups who often face language barriers and differing cultural health beliefs (30). This narrow clinical focus may limit the applicability of findings in real-world, community-based settings, where multiple factors influence health behaviours and outcomes (9, 12).

The implications for policy and practice are considerable. First, there is an urgent need for public health policies to actively promote inclusive research practices. This includes mandating the collection and disaggregation of ethnicity data in clinical trials and requiring the inclusion of diverse populations in funded research studies. These measures would not only improve the evidence base but also help reduce health disparities. For clinical practice, healthcare providers should be trained in cultural competence and encouraged to adopt communication strategies that cater to linguistically diverse patients. The success of bilingual helplines and peer-led education in the reviewed studies points to the value of culturally congruent care models (12, 31).

Additionally, commissioning bodies should invest in interventions that are community-based and co-designed with service users from Black, Asian and Minority Ethnic groups. Peer education models, for instance, were shown to improve health literacy and engagement among South Asian patients with RA and those at risk of rickets (20, 22, 27). These community-driven approaches can increase trust and ensure the relevance and sustainability of health interventions. Moreover, structural factors such as delayed referrals, systemic biases in healthcare access, and socioeconomic disparities need to be addressed through integrated service delivery and cross-sector collaboration (32, 33).

From a research perspective, this review highlights critical gaps. Few studies employed participatory or qualitative methodologies to understand the lived experiences of Black, Asian and Minority Ethnic individuals with MSDs. Research that centres the voices of marginalised communities can offer more nuanced insights into barriers to care, treatment adherence, and preferences for intervention delivery. Further, the predominance of quantitative clinical research risks neglecting the social determinants of health that underpin disparities in MSD outcomes. Future research should adopt a more holistic approach, combining epidemiological studies with community-based participatory research, to inform interventions that are both clinically effective and socially appropriate.

One of the key strengths of this scoping review is its systematic methodology, incorporating a comprehensive database search and rigorous screening process aligned with the PCC framework. The inclusion of both qualitative and quantitative studies allowed for a broader thematic analysis of interventions, while the narrative synthesis approach enabled integration across diverse research designs. Additionally, the review’s UK-specific context ensured relevance to current NHS practices and health equity goals. However, limitations must be acknowledged. First, the reliance on peer-reviewed literature published in English may have excluded relevant studies available in other languages or grey literature, such as community evaluations or NHS reports. Second, the review included only studies that stratified data by ethnicity, potentially omitting valuable insights from broader studies where ethnicity-specific findings were not reported. This highlights an ongoing issue in research reporting, where ethnic subgroup analysis is often absent or insufficiently detailed. Lastly, the limited number of studies that met the inclusion criteria restricts the generalisability of the findings and underscores the need for more comprehensive and inclusive research moving forward.

## Conclusion

This scoping review highlights that despite the disproportionate burden of MDSs among Black, Asian and Minority Ethnic populations in the UK, there is limited scope for interventions designed to address the specific needs of Black, Asian and Minority Ethnic groups with MSDs. Most existing studies are clinically focused and lack ethnic inclusivity. Notably, within the Black, Asian and Minority Ethnic category, there appears to be a consistent omission of the Black African and Black Caribbean groups living in the UK. Policymakers, practitioners, and researchers must work collaboratively to co-design culturally tailored, community-based interventions and prioritise the inclusion of ethnically diverse participants in MSD research. Only through such concerted efforts can we begin to bridge the gap in health outcomes and ensure equitable care for all.

## Data Availability

The original contributions presented in the study are included in the article/Supplementary material, further inquiries can be directed to the corresponding author.

## Footnotes

^1^ https://www.rayyan.ai

^2^ www.lumivero.com
