# Supplementary material for: Interventions supporting people from Black, Asian and Minority Ethnic groups in the United Kingdom with musculoskeletal disorders: a scoping review
Source: Front Public Health. 2025 Oct 2;13:1675860. doi: 10.3389/fpubh.2025.1675860 (PMC12528078; doi:10.3389/fpubh.2025.1675860)
Supplement: Supplementary file 1 [file Data_Sheet_1.docx]

**Supplementary file 1**

**CINAHL**

1. "South Asian*" OR "Asian" OR "Black Africans" OR "black Caribbean" OR "African*" OR “Culturally and linguistically diverse” OR "CALD" OR "Afro-Caribbean" OR "ethnic groups" OR "minority" OR "ethnic minority" OR "ethnic inequalities" OR "BAME" OR "migrants" OR "diverse communities" OR "diversity" OR "cultural sensitivity" OR "racist" OR "multicultural" OR "ethno-cultural" OR "ethnocentric" OR "non-white" OR "deprived" OR "urban communities" OR "Underserved" OR Cultur* OR (MM "Linguistic Minorities") OR (MH "Ethnic Groups+") OR (MH "Minority Groups+") OR (MM "Migrants") OR (MH "Cultural Values+") OR (MH "Cultural Diversity+")
2. “musculoskeletal health” OR “musculoskeletal disorder” OR “upper extremity” OR “non-malignant pain” OR “neck-shoulder pain” OR “muscle pain” OR “elbow pain” OR “hip pain” OR “hand pain” OR “musculoskeletal” OR “upper limb” OR “lower limb” OR “MSDs” OR “spinal pain” OR “ankle pain” OR “Bone*” OR “Lupus” OR “MSK” OR “coxa” OR “knee pain” OR “foot pain OR (MH "Musculoskeletal Diseases+")
3. “Intervention” OR “ergonomic” OR “training” OR “supervisor” OR “effect*” OR “educational program” OR “prevention” OR “protect” OR “evaluation*” OR “Healthcare” OR “Physical activity” OR “Support” OR “Access” OR “working alliance” OR “Health capital” OR “Connections” OR “person-centred” OR “workplace-based intervention” OR “evaluation*” OR “training” OR “educational program” OR “patient education” OR “educat*” OR “physical*” OR “mental*” OR “emotional*” OR “care*” OR “Primary care” OR “Patient centred” OR “aerobic exercise” OR “pedometers” OR “step counter” OR “computer-assisted” OR “decision support system” OR “knowledge base” OR “vocational” OR “work*” OR “work capacity” OR “job*” OR “employee” OR “staff” OR “personnel” OR “outdoor work*” OR “day shift*” OR “support group” OR “night shift*” OR “peer support*” OR “vocational reintegration” OR (MH "Experimental Studies+") OR (MM "Intervention Trials") OR (MH "Randomized Controlled Trials+") OR (MH "Health Education+") OR (MM "Peer Assistance Programs") OR (MM "Community Programs") OR (MH "Exercise+")
4. "UK" OR "United Kingdom" OR "England" OR "Wales" OR "Northern Ireland" OR "Britain" OR "Scotland" OR (MH "United Kingdom+")
5. 1 AND 2 AND 3 AND 4 **[1012 Studies]**

**PsycINFO**

1. (DE "Ethnic Identity") OR (DE "Ethnolinguistics") OR "South Asian*" OR "Asian" OR "Black Africans" OR "black Caribbean" OR "African*" OR “Culturally and linguistically diverse” OR "CALD" OR "Afro-Caribbean" OR "ethnic groups" OR "minority" OR "ethnic minority" OR "ethnic inequalities" OR "BAME" OR "migrants" OR "diverse communities" OR "diversity" OR "cultural sensitivity" OR "racist" OR "multicultural" OR "ethno-cultural" OR "ethnocentric" OR "non-white" OR "deprived" OR "urban communities" OR "Underserved" OR Cultur*
2. (DE "Musculoskeletal Disorders") OR (MH "Experimental Studies+") OR “musculoskeletal health” OR “musculoskeletal disorder” OR “upper extremity” OR “non-malignant pain” OR “neck-shoulder pain” OR “muscle pain” OR “elbow pain” OR “hip pain” OR “hand pain” OR “musculoskeletal” OR “upper limb” OR “lower limb” OR “MSDs” OR “spinal pain” OR “ankle pain” OR “Bone*” OR “Lupus” OR “MSK” OR “coxa” OR “knee pain” OR “foot pain
3. “Intervention” OR “ergonomic” OR “training” OR “supervisor” OR “effect*” OR “educational program” OR “prevention” OR “protect” OR “evaluation*” OR “Healthcare” OR “Physical activity” OR “Support” OR “Access” OR “working alliance” OR “Health capital” OR “Connections” OR “person-centred” OR “workplace-based intervention” OR “evaluation*” OR “training” OR “educational program” OR “patient education” OR “educat*” OR “physical*” OR “mental*” OR “emotional*” OR “care*” OR “Primary care” OR “Patient centred” OR “aerobic exercise” OR “pedometers” OR “step counter” OR “computer-assisted” OR “decision support system” OR “knowledge base” OR “vocational” OR “work*” OR “work capacity” OR “job*” OR “employee” OR “staff” OR “personnel” OR “outdoor work*” OR “day shift*” OR “support group” OR “night shift*” OR “peer support*” OR “vocational reintegration”
4. (MH "United Kingdom+") OR "UK" OR "United Kingdom" OR "England" OR "Wales" OR "Northern Ireland" OR "Britain" OR "Scotland"
5. 1 AND 2 AND 3 AND 4 **[126 studies]**

**Web of Science**

1. [All fields] "South Asian*" OR Asian* OR "Black African" OR "black Caribbean" OR "African*" OR "Afro-Caribbean" OR "minority" OR BAME OR migrants OR cultur*
2. [All fields] "musculoskeletal disorder" OR pain OR "musculoskeletal" OR "MSK"
3. [All fields] Intervention* OR Trial OR ergonomic OR education* OR aware* OR prevent* OR rehabilitation OR palliative OR work* OR exercise*
4. "UK" OR "England" OR "Britain" OR Scotland OR Wales OR "United Kingdom" [All fields, Abstract]
5. 1 AND 2 AND 3 AND 4 **[518 studies]**

**Scopus**

1. [ALL] "South Asian*" OR "Asian" OR "Black Africans" OR "black Caribbean" OR "African*" OR “Culturally and linguistically diverse” OR "CALD" OR "Afro-Caribbean" OR "ethnic groups" OR "minority" OR "ethnic minority" OR "ethnic inequalities" OR "BAME" OR "migrants" OR "diverse communities" OR "diversity" OR "cultural sensitivity" OR "racist" OR "multicultural" OR "ethno-cultural" OR "ethnocentric" OR "non-white" OR "deprived" OR "urban communities" OR "Underserved" OR Cultur*
2. [ALL] “musculoskeletal health” OR “musculoskeletal disorder” OR “upper extremity” OR “non-malignant pain” OR “neck-shoulder pain” OR “muscle pain” OR “elbow pain” OR “hip pain” OR “hand pain” OR “musculoskeletal” OR “upper limb” OR “lower limb” OR “MSDs” OR “spinal pain” OR “ankle pain” OR “Bone*” OR “Lupus” OR “MSK” OR “coxa” OR “knee pain” OR “foot pain
3. [ALL] “Intervention” OR “ergonomic” OR “training” OR “supervisor” OR “effect*” OR “educational program” OR “prevention” OR “protect” OR “evaluation*” OR “Healthcare” OR “Physical activity” OR “Support” OR “Access” OR “working alliance” OR “Health capital” OR “Connections” OR “person-centred” OR “workplace-based intervention” OR “evaluation*” OR “training” OR “educational program” OR “patient education” OR “educat*” OR “physical*” OR “mental*” OR “emotional*” OR “care*” OR “Primary care” OR “Patient centred” OR “aerobic exercise” OR “pedometers” OR “step counter” OR “computer-assisted” OR “decision support system” OR “knowledge base” OR “vocational” OR “work*” OR “work capacity” OR “job*” OR “employee” OR “staff” OR “personnel” OR “outdoor work*” OR “day shift*” OR “support group” OR “night shift*” OR “peer support*” OR “vocational reintegration”
4. [ALL] "UK" OR "United Kingdom" OR "England" OR "Wales" OR "Northern Ireland" OR "Britain" OR "Scotland" **[1636 studies]**
